# Identifying persistent negative symptoms in first episode psychosis

**DOI:** 10.1186/1471-244X-12-224

**Published:** 2012-12-06

**Authors:** Cindy L Hovington, Michael Bodnar, Ridha Joober, Ashok K Malla, Martin Lepage

**Affiliations:** 1 Prevention and Early Intervention Program for Psychoses (PEPP-Montreal), Douglas Mental Health University Institute, Montreal, Quebec, Canada; 2 Department of Neurology & Neurosurgery, Montreal Neurological Institute, McGill University, Montreal, Quebec, Canada; 3 Department of Psychiatry, McGill University, Montreal, Quebec, Canada; 4 Douglas Mental Health University Institute, Frank B Common Pavilion, F1143, 6875 LaSalle Blvd, Verdun, Quebec, H4H 1R3, Canada

**Keywords:** First-episode psychosis, Persistent negative symptoms, Negative symptoms, Functional outcome

## Abstract

**Background:**

Although persistent negative symptoms (PNS) are known to contribute significantly to poor functional outcome, they remain poorly understood. We examined the heuristic value of various PNS definitions and their respective prevalence in patients with first episode psychosis (FEP). We also contrasted those definitions to the Proxy for the Deficit Syndrome (PDS) to identify deficit syndrome (DS) in the same FEP cohort.

**Methods:**

One hundred and fifty-eight FEP patients were separated into PNS and non-PNS groups based on ratings from the Scale for Assessment of Negative Symptoms (SANS). PNS was defined in the following ways: 1) having a score of 3 or greater on at least 1 global subscale of the SANS (PNS_1); 2) having a score of 3 or more on at least 2 global subscales of the SANS (PNS_2); and 3) having a score of 3 or more on a combination of specific SANS subscales and items (PNS_H). For all three definitions, symptoms had to be present for a minimum of six consecutive months. Negative symptoms were measured upon entry to the program and subsequently at 1,2,3,6,9 and 12 months. Functional outcome was quantified at first assessment and month 12.

**Results:**

PNS prevalence: PNS_1 (27%); PNS_2 (13.2%); PNS_H (13.2%). The prevalence of DS was found to be 3% when applying the PDS. Regardless of the definition being applied, when compared to non-PNS, patients in the PNS group were shown to have significantly worse functioning at month 12. All three PNS definitions showed similar associations with functional outcome at month 12.

**Conclusion:**

Persistent negative symptoms are present in about 27% of FEP patients with both affective and non-affective psychosis. Although there has previously been doubt as to whether PNS represents a separate subdomain of negative symptoms, the current study suggests that PNS may be more applicable to FEP when compared to DS. Although all three PNS definitions were comparable in predicting functional outcome, we suggest that the PNS definition employed is dependent on the clinical or research objective at hand.

## Background

Growing evidence has suggested that negative symptoms in psychotic disorders are intractable and associated with poor functional outcome [1-3]. According to the most recent National Institute of Mental Health (NIMH) consensus statement, the negative symptom construct includes blunted affect, anhedonia, alogia, asociality and avolition [4]. However, broadly classifying negative symptoms into 5 categories does not take into account etiology and duration, which contribute to the heterogeneity of these symptoms [5]. Thus, negative symptoms are further subdivided into the following subtypes: 1) primary or idiopathic negative symptoms, 2) secondary negative symptoms (caused by positive symptoms, depression, or extrapyramidal symptoms), 3) deficit syndrome or deficit schizophrenia (DS), believed to be a pathophysiological distinct disease within schizophrenia and is diagnosed based on the presence of primary enduring (minimum of 12 consecutive months) [6], and 4) persistent negative symptoms (PNS) (primary or secondary negative symptoms evident for 6 consecutive months after the stabilization of a first episode of psychosis) [5].

Persistent negative symptoms have become a major concern given their resistance to treatment and persistence throughout the illness, leading to poor prognosis. Varying terminology and criteria have been used to describe and identify PNS. Consequently, this lack of a consensus definition has yielded mixed results in terms of structural, neuropsychological and functional correlates of PNS [for review see [7]]. Recently, Buchanan suggested that the duration and severity of negative symptoms must be taken into account when identifying PNS. The following criteria were proposed: having at least moderate negative symptoms, having negligible positive, depressive or extrapyramidal symptoms, and clinical stability for an extended period of time [5]. Empirical evidence on the proposed criteria for PNS has been scant.

Some have suggested that PNS may represent a broader concept than deficit syndrome [5,8]. Deficit syndrome, which is proposed to identify a putatively more homogenous subgroup in schizophrenia, highlights the manifestation of prominent, primary and enduring negative symptoms that are resistant to treatment. The criteria for DS requires that negative symptoms of significant severity be present for a minimum of one year, to have been present at baseline (during periods of relative remission) and are not secondary in nature [6]. Furthermore, patients must meet the DSM criteria for schizophrenia spectrum disorder [6]. Deficit syndrome is assessed using the Schedule for the Deficit Syndrome (SDS), which is a semi-structured interview measuring the persistence of 6 negative symptoms including restricted affect, diminished emotional range, poverty of speech, curbed interests, diminished sense of purpose, and diminished social drive [6]. An individual must have moderate to severe scores on at least 2 of these 6 symptoms. After the introduction of the SDS, the Proxy for the Deficit Syndrome (PDS) was introduced as a case identification for measuring deficit symptoms [9]. This tool allows one to administer common negative symptoms scales such as the Positive and Negative Syndrome Scale (PANSS) or Brief Psychiatric Rating Scale (BPRS) and to apply the PDS formula to obtain a score determining whether the patient meets the criteria for DS. The PDS is defined as the sum of the scores for Anxiety, Guilt Feelings, Depressive Mood and Hostility items from the scales subtracted from the score of Blunted Affect [9].

Unlike DS which is quantified using the SDS [6], negative symptom severity for PNS can be measured using any validated negative symptom scale. According to the NIMH consensus statement [4] the Scale for the Assessment of Negative Symptoms (SANS) [10] has the most extensive coverage. Other scales such as the PANSS [11] are also widely used, but do not provide as much detail on negative symptoms as the SANS. However, some concerns regarding these scales have been raised. For instance, early evidence suggests that some items from the SANS including, “poverty of content of speech” and “inappropriate affect” represent a disorganization dimension rather than negative symptoms of schizophrenia [12]. Exploratory and confirmatory factor analytical studies identified three underlying factors in the negative symptoms construct including 1) affective flattening 2) avolition/apathy and anhedonia/asociality and 3) inattention/alogia [13-15]. Concordant factors have been documented in a FEP cohort [16]. These factors are incorporated into the SANS [10]. However, there is now a general consensus that inattention may not be conceptually related to negative symptoms [8,17,18]. Furthermore, some findings are suggestive of interrelated yet separate subdomains of negative symptoms in schizophrenia including, 1) diminished expression, composed of affective flattening and poverty of speech, and 2) amotivation, consisting of avolition/apathy and anhedonia/asociality [4,8,17]. Similarly, in patients with DS, a principle component analysis using the Schedule for Deficit Syndrome indicated that DS is best described by two factors including avolition and reduced emotional expression [19]. It is possible that this multidimensionality within negative symptoms is relevant not only to chronic schizophrenia but to FEP patients with PNS as well; this has not been investigated.

The lack of “gold standard” for PNS has brought up some major concerns [4,20-22]. Also, studies have not employed comparable criteria to identify PNS. For instance, while one study included patients in the “negative symptom group” if they scored 2 or more on a minimum of 1 global SANS subscales [23], others have used a score of 3 or more [3]. In addition, some have also applied criteria that involve having clinically significant symptoms (score ≥3) on a minimum of 2 global items of the SANS [24].

Given this variability, it is likely that using different criteria for identifying PNS will yield mixed results. Hence, there is a need for PNS criteria that are clinically useful in identifying PNS. The first episode of psychosis may be a critical time to identify individuals with PNS in order to potentially influence these symptoms through more focused intervention such as intensive psychosocial interventions. Further, given the lack of consensus definition for PNS, its prevalence in FEP using well-defined criteria remains unknown. The main objective of this paper was to examine the heuristic value of various PNS definitions and their respective prevalence in patients with first episode psychosis. Second, given that DS also represents a subgroup of patients with enduring negative symptoms, we wanted to contrast the PNS definitions with the proxy definition for deficit syndrome in a FEP cohort. To substantiate the clinical predictive validity of the abovementioned definitions, all were explored in association with patient function followed over a 12-month period in a cohort of first-episode of psychosis patients. We hypothesize that patients meeting the PNS criteria will have poorer functioning than those not meeting the criteria [25-27].

## Methods

### Subjects

All patients were part of a longitudinal naturalistic outcome study of first-episode psychosis and were recruited and treated through the Prevention and Early Intervention Program for Psychoses (PEPP-Montreal), a specialized early intervention service with integrated clinical, research, and teaching modules, at the Douglas Mental Health University Institute in Montreal, Canada. Individuals aged 14 to 35 years from the local catchment area suffering from either affective or non-affective psychosis that had not taken antipsychotic medication for more than one month and with an IQ higher than 70 were consecutively admitted to the program as either in- or out-patients. For complete program details see Malla et al. [28] or visit http://www.douglasresearch.qc.ca/pages/view?section_id=165. Patients were diagnosed according to DSM-IV criteria using the Structured Clinical Interview for DSM-IV (SCID-IV) [29]. Written informed consent was obtained from all participants. Research protocols were approved by the Douglas Institute Human Ethics Review Board.

### Clinical assessment

For all subjects who met the inclusion criteria, an initial assessment was conducted on average, within one month after admission (in days; mean=22.7, s.d.= 8.6, range=8.3-54.8). At the initial assessment the following data were acquired: education level (number of school years completed), Full Scale IQ with the Wechsler Adult Intelligence Scale [30], parental socio-economic status (SES) with the Hollingshead two-factor index [31], The Premorbid Adjustment Scale (PAS) [32], and handedness [33]. Negative and positive symptoms were assessed with the Positive and Negative Symptoms Scale (PANSS) as well as the SANS [10] and SAPS [34], respectively. The domain of attention in the SANS scale was not included in our analyses. Evaluators at PEPP established an ICC of 0.74 on the SAPS and 0.71 on the SANS; all raters participated in inter-rater reliability sessions at least once a year to avoid rater drift (i.e. raters must maintain consistency with themselves as well as with other raters). Depressive symptoms were assessed with the Calgary Depression Scale for Schizophrenia (CDSS) [35] and extrapyramidal symptoms with the Extrapyramidal Symptoms Rating Scale (ESRS) [36]. If prescribed, based on the ESRS and attending physician’s discretion, type and dose of anticholinergic taken were recorded. The type and dosage of antipsychotics taken were also recorded and subsequently converted into chlorpromazine equivalents [37]. As part of the longitudinal study, severity of positive, negative, depressive and extrapyramidal symptoms was evaluated at initial assessment and 1,2,3,6,9 and 12 months later, using the SAPS, SANS, CDS and ESRS, respectively.

The period of “prodrome”, calculated through the Circumstances of Onset and Relapse Schedule (CORS) interview which is based on the Interview for the Retrospective Assessment of Schizophrenia (IRAOS) [38], was defined as the time between the onset of any psychiatric symptoms and the onset of the presenting psychotic episode. From this interview, such variables as duration of untreated psychosis (DUP), duration if untreated illness (DUI), pre-morbid functioning levels, and socio-economic status are obtained. Psychiatric symptoms refer to symptoms indicating a behavioural change such as anxiety, depression, suicidal ideation, or social withdrawal as well as sub-threshold psychotic symptoms such as suspiciousness and odd ideas and behaviour and do not include developmental disorders. Duration of untreated psychosis was calculated as time from the first episode to the date of entry into the program. Finally, DUI was calculated as the time between the first ever onset of any psychiatric symptoms to the time of adequate treatment, as above [39]. Duration of untreated illness included periods of psychiatric symptoms not necessarily contiguous with the psychotic episode and interspersed with relatively healthy periods. All other demographic data were obtained through the same interview.

### Method for identifying persistent negative symptoms

Upon completing 12 months of the treatment program, clinical data were analyzed and PNS definitions were applied based on data collected from the first assessment, months 1,2,3,6,9 and 12. Negative symptoms were required to be present after the initial stabilization of symptoms (month 3) and maintained for 6 consecutive months (months 6, 9 and 12) with at least a moderate severity as measured on a validated scale [5]. Although 6 months has often been employed as the point of initial stabilization [24], our previous findings along with recent data in FEP, suggests a decrease of acute psychotic symptoms and an initial stabilization period closer to 3 months [40-43]. In addition, factor analytical studies have suggested that some items of the SANS including, “poverty of content of speech” and “inappropriate affect” poorly correlate with the scale [13,15]. Hence, as suggested by Malla et al. [3], if the global rating on “affective flattening” or “alogia” was based entirely as a result of items “inappropriate affect” or “poverty of content of speech”, respectively, such patients were not included in the PNS group.

All subjects with secondary negative symptoms were excluded from analyses. Patients were required to have a global rating of mild (2) or less on all positive symptoms as measured by the Scale for the Assessment of Positive Symptoms (SAPS) [34], a total score of 4 or less on the Calgary Depression Scale for Schizophrenia (CDSS) [35], and extrapyramidal symptoms that were absent or too mild to require treatment with anticholinergic medication based on the ESRS [36].

All of the above mentioned criteria had to be maintained for a period of at least 6 consecutive months (specifically between month 6 and 12 after admission). In addition to having negative symptoms for 6 consecutive months, patients with affective or non-affective psychosis were also required to have clinically significant negative symptoms (“clinically significant” symptoms were considered to be moderate to severe scores on SANS items, or scores of 3 or greater) on the SANS scale at month 3.

Lastly, three PNS definitions applied in various studies for identifying persistent negative symptoms during a 12-month longitudinal study and the PDS for identifying DS were explored [3,9,24]:

#### Persistent negative symptom

1. PNS_1: a score of 3 or more on *at least 1 global item* of the SANS [3].

2. PNS_2: a score of 3 or more on *at least 2 global items* of the SANS [24].

3. PNS_H: a SANS score of 3 or more on either one or both of the following subdomains as previously described by Foussias and Remington [8]: 1) *Diminished expression* (must have a score of 3 or more for both affective flattening and poverty of speech) and/or 2) *Amotivation* (must have a score of 3 or more for both avolition/apathy and anhedonia/asociality).

#### Deficit syndrome

1. DS (using PANSS): PDS Score = Blunted Affect (n1) – [Anxiety (G2) + Guilty Feelings (G3) + Depressed Mood (G6) + Hostility Items (P7)]. In order to be classified as meeting the criteria for DS, patients were required to have a score greater than two on the PDS [9]. Similarly to PNS, this criterion had to be met at months 3,6,9 and 12. In other words, the proxy definition was not employed for 12 consecutive months, as it is required. Given that this study examined a group with FEP, retrospectively assessing DS would have begun at the first episode when symptoms are not yet stable.

### Statistical analysis

All analyses were conducted using PASW version 18 (SPSS, Chicago, IL) and were two-tailed with a critical *p*-value of 0.05. Group differences with regard to the first assessment variables for DUP, DUI, length of prodrome and SOFAS scores were analyzed using independent t-tests. The following clinical characteristics were not normally distributed (Shapiro-Wilks W test): DUP, DUI prodrome and CDSS scores. Prodrome, DUI and CDSS scores were normalized using square root transformations, while DUP was normalized using a logarithmic transformation. Group differences were also compared at several time points (first assessment, months 3 and 12) for clinical symptoms including SANS, SAPS and CDSS total scores as well as for SOFAS scores using independent t-tests. Independent t-tests were also used to compare group differences between PNS and non-PNS for age, DUI, DUP and prodrome. The prevalence of PNS in patients with FEP was determined at month 12. Patients were categorized into PNS or non-PNS (and further subdivided according to which PNS criteria they met). In addition, patients who met the PDS criteria according to the previously published cutoff (≥2) [9] were categorized in the DS group. To determine the association between PNS and function, repeated measures ANOVA were used to examine group differences between PNS definitions (each definition separately) and SOFAS score (first assessment, month 12) used as the within subject variable and group (PNS, Non-PNS) as the between subject variable.

## Results

### Demographics and symptoms

**Figure 1:**
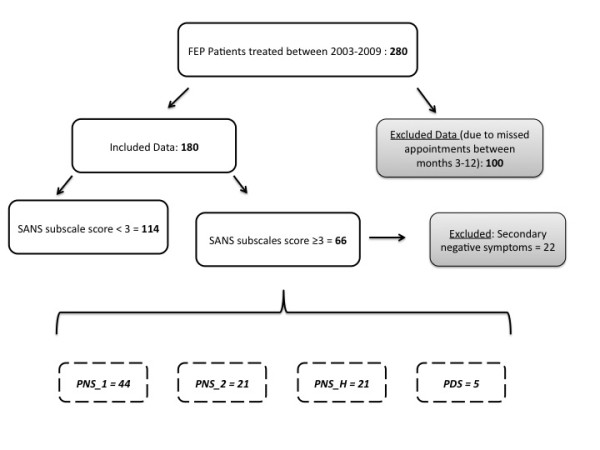
Classification of FEP patients based on negative symptoms.

Data from a cohort of 280 FEP patients treated between 2003 and 2009 were collected. Of these, 100 had missing clinical data between months 3 and 12 due to one or more missed assessments. These subjects were excluded from further analyses since they could not be classified as PNS or non-PNS. Of note, no differences in age, DUP, DUI or prodrome were found between included patients and excluded patients due to missing data. Sixty-six of the 180 FEP patients had a score greater or equal to 3 on at least one SANS subscales. Twenty-two out of these 66 patients were excluded because of secondary negative symptoms (15 for moderate positive symptoms with SAPS score >2, six had clinically significant depressive symptoms, 3 for extrapyramidal symptoms and 1 for substance-induced symptoms (Note: two patients met the criteria for more than one secondary negative symptom). Hence, 44 FEP patients were included in the PNS group for analyses. Figure 1 illustrates patient classification for the current study.

**Figure 2:**
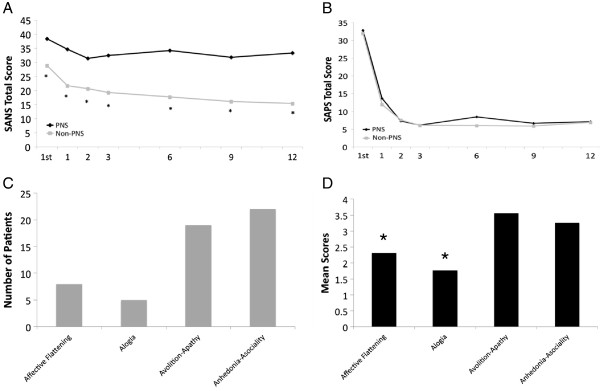
A) SANS total scores from first assessment to month 12; B) SAPS total scores from first assessment to month 12 *= p<0.001; C) Frequency of patients that met PNS_**1 criteria for the 4 subdomains of the SANS; D) Mean scores for the 4 subdomains of the SANS for patients meeting the PNS**_**1 criteria.** N= 158 (44 PNS and 114 non-PNS) *= Domain was significantly different than all the other three SANS domains. *1*^*st*^= First Assessment. *Note*: SANS total scores do not include the “Attention” domain.

See Table 1 for results of patient demographics and clinical characteristics. Patients with PNS did not differ from patients without PNS in age, gender, DUI, DUP, and prodrome. The PNS group had significantly worse functioning at first assessment and at month 12 when compared to non-PNS groups. The PNS group had significantly worse negative symptoms scores than non-PNS at all time points. The PNS and non-PNS groups had similar scores on both positive symptom and depression scales. Mean negative and positive symptom scores for PNS and non-PNS were compared at each assessment and are presented in Figure 2A and B. Similar to previous findings [40-43], we also documented an initial stabilization of symptoms by the third month of treatment. Individual domains were explored (Figure 2C and D). The frequency of patients with PNS who met the criteria (≥3) for any of the 4 domains SANS as well as the mean score of the PNS group for each domain was explored. Patients meeting the criteria for PNS_1 had higher levels of avolition/apathy as well as anhedonia/asociality both in terms of meeting the PNS criteria due to these domains as well as having higher mean scores in these domains. Primary diagnoses for all patients in the PNS groups are found in Table 2. The majority of patients in the PNS_1 group were diagnosed with schizophrenia (paranoid) (20.5%) or schizophrenia (undifferentiated) (20.5%). On the other hand, a greater number of patients in the PNS_2 and PNS_H groups were diagnosed with schizoaffective disorder or schizophrenia (undifferentiated).

**Table 1:** **Demographics and clinical characteristics of PNS cohort** (**as per PNS**_**1 criteria**)

	***PNS***	***Non***-***PNS***	***F***, ***χ ***^***2***^***or t***,
	**(n=44)**	***(n=114)***	***p***-***value***
Age at entry, years	22.0±4.0	22.9±4.1	t=1.29, .195
Gender (m/f)	30/14	82/32	*χ*^*2*^ =0.216, .697
DUI, weeks ^b^	286.9±273.3	254.7±260.1	t=−0.679, .498
DUP, weeks ^b^	42.3±56.3	47.7±110.4	t=0.305, .761
Prodromal period ^b^	104.3±178.3	90.1±159.2	t= −0.478, .634
**SOFAS**			
1^st^ assessment	40.0±12.7	47.0±14.5	t=2.403, .018*
Month 12	54.2±14.9	67.5±14.4	t=4.233, <.001*
**SANS Total**			
1^st^ assessment	40.0±17.6	27.5±15.5	t=−4.335, <.001*
Month 3	33.1±13.0	18.6±11.5	t=−6.594, <.001*
Month 12	33.7±14.5	15.6±11.1	t=−7.961, <.001*
**SAPS Total**			
1^st^ assessment	31.2±12.4	31.6±13.7	t=−0.153, .878
Month 3	6.7±7.2	4.2±5.4	t=−2.320, .047*
Month 12	6.6±6.9	6.8±12.4	t= .103, .918
**CDSS Total**			
1^st^ assessment	5.3±4.8	4.9±5.3	t=−0.347, .729
Month 3	1.9±3.0	2.0±3.1	t=0.296, .768
Month 12	1.6±2.8	1.5±2.9	t=−0.314, .754

DUP= duration of untreated psychosis (from first episode to date of entry to PEPP); DUI=duration of untreated illness; SANS= Scale for the Assessment of Negative Symptoms; SAPS= Scale for the Assessment of Positive Symptoms; SOFAS = Social and Occupational.

^a^ Hollingshead Parental Socio-Economic Status, in which 1 = highest and 5 = lowest.

^b^ Analyses were made with transformed data but values are presented in raw form.

Note: Secondary negative symptoms were removed from analyses.

Data presented as mean ± SD (*χ*^*2*^or t, p-value).

**Table 2:** Primary diagnosis on admission

**Diagnosis**	**PNS**_**1**	**PNS**_**2**	**PNS**_**H**	**PDS**
	***N=44***	***N=21***	***N=21***	***N=5***
Schizophrenia-Disorganized	4 (9.1)	2 (9.5)	2 (9.5)	1 (20.0)
Schizophrenia- Paranoid	*9* (*20*.*5*)	*3* (*14*.*3*)	*3* (*14*.*3*)	1 (20.0)
Schizophreniform	1 (2.3)	-	-	-
Schizoaffective	*8* (*18*.*2*)	*7* (*33*.*3*)	*7* (*33*.*3*)	-
Schizophrenia- Undifferentiated	*9* (*20*.*5*)	*6* (*28*.*6*)	*6* (*28*.*6*)	*3* (*60*.*0*)
Bipolar I – With psychotic features	5 (11.4)	-	-	-
Major Depression – With psychotic features	2 (4.5)	1 (4.8)	1 (4.8)	-
Bipolar I – Manic with psychotic features	2 (4.5)	1 (4.8)	1 (4.8)	-
Bipolar I – Depressed recent episode	1 (2.3)	1 (4.8)	1 (4.8)	-
Psychosis NOS	3 (6.8)	-	-	-

Data presented as frequency (percentage).

#### Prevalence and associations with functional outcome

##### PNS_1 definition

**Figure 3:**
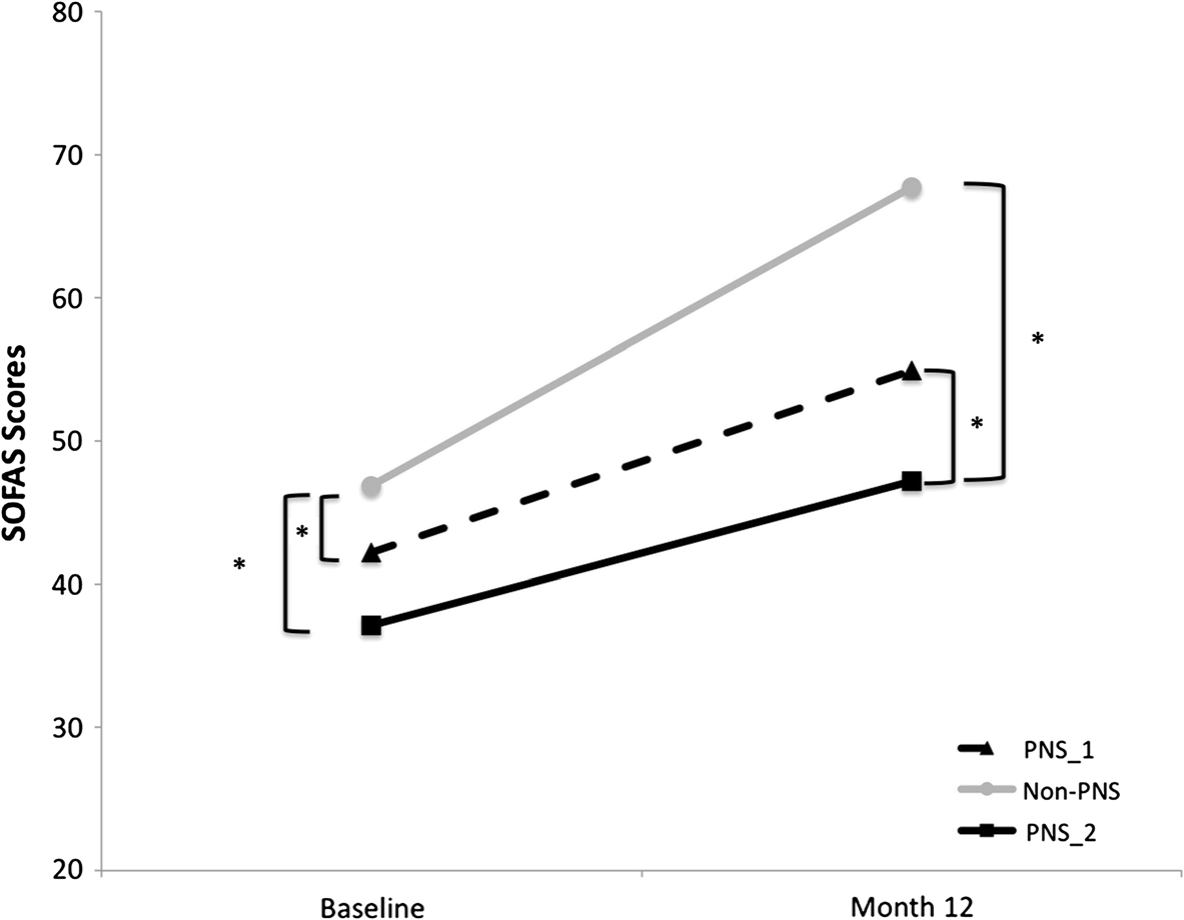
**Mean SOFAS scores at the first assessment and month 12 follow**-**up for patients with and without PNS.**

Forty- four patients (27.8%) were identified with PNS and 114 (72.2%) were not. This liberal definition showed the highest prevalence of PNS compared to the other two definitions. The repeated measures ANOVA revealed a significant main effect of time (F_1,82_=71.762, p<0.001) and a significant main effect of group (F_1,82_=10.065, p=0.002). Figure 3 illustrates SOFAS scores over time for PNS_1, PNS_2 and non-PNS patients. A significant [time x group] interaction (F_1,83_=4.117, p=0.046) was also observed. Further analyses revealed SOFAS scores for patients with PNS were significantly lower at both initial assessment (F_1,125_=4.343, p=0.039) and at month 12 (F_1,109_=17.328 p<0.000) compared to patients without PNS. Paired t-tests revealed that PNS_1 (t_23_=−4.335, p<0.000) and non-PNS (t_59_= −9.414, p<0.000) both significantly improved over time. A score of 60 or greater on the SOFAS is considered to represent good functioning [44,45]. Interestingly, only the non-PNS group had a mean score greater than 60 on the SOFAS scale at month 12. See Table 1 for SOFAS scores.

##### PNS_2 definition

Twenty-one patients (13.3%) met the criteria for PNS_2, while 137 did not (86.7%). Similarly to the PNS_1 definition, repeated measures ANOVA revealed a significant main effect of time (F_1,82_=28.525, p<0.001) and a significant effect of group (F_1,82_=14.661, p<0.001). There was no significant [time x group] interaction (F_1,82_=2.956, p=0.089) observed. Further analyses revealed that patients with PNS had significantly worse functioning at the initial assessment (F_1,126_= 6.669, p=0.011) and at month 12 (F_1,103_ =20.981, p<0.001) than patients in the non-PNS group. Paired t-test revealed that PNS_2 (t_9_=−2.239, p<0.052) and non-PNS (t_73_= −10.072, p<0.000) both significantly improved function over time. Similarly to the PNS_1 definition, the mean SOFAS score for the PNS_2 group was not greater than 60, which is considered to be “poor functioning”.

##### PNS_H definition

Twenty-one patients (13.3%) met the criteria for PNS_H, while 137 (86.7%) did not. Repeated measures ANOVA revealed a significant main effect of time (F_1,82_=28.525, p<0.001) as well as a significant effect of group (F_1,82_=14.661, p<0.001). No significant [time x group] interaction was found (F_1,82_=2.956, p=0.089). Group comparisons with a one-way ANOVA revealed significantly worse SOFAS scores for the PNS group at the initial assessment (F_1,126_=6.669, p=0.001) and at month 12 (F_1,102_=20.981, p<0.001). Paired t-tests revealed that both PNS (t_9_=−2.239, p<0.052) and non-PNS (t_73_= −10.072, p<0.000) groups improved in function over time. Only patients not meeting the PNS_H criteria achieved good functioning.

##### Prevalence of DS in FEP

Only five FEP patients (3%) met the criteria for deficit syndrome according to the proxy definition. All five patients in this group had an initial diagnosis of schizophrenia (disorganized, paranoid or undifferentiated).

Furthermore, as previously mentioned, when compared to any of the 3 PNS groups, only the cohort with non-PNS patients achieved a mean score of “good functioning” according to the SOFAS scale. Due to missing data, only 140 of the patients obtained a SOFAS score at month 12 (31 from the PNS cohort and 109 from the non-PNS cohort). However, of those with available SOFAS scores at the 12 month follow-up, 61% of patients from the PNS_1 group (19/31) were considered “poorly functioning” whereas only 26% of non-PNS patients (28/109) were “poor functioning”. Interestingly, of the 39% of PNS patients (12/31) with “good functioning”, 83% (10/12) met the PNS criteria for PNS_1 only and not PNS_2 or PNS_H.

#### Supplementary analysis of the PNS_1 group

To further delineate whether a stringent PNS definition is more clinically significant, “liberal” and “stringent” subgroups were formed. More specifically, the “stringent” group was formed of both PNS_2 and PNS_H groups combined. To obtain a “clean” PNS_1 group, specific patients in the PNS_1 group were extracted and re-named as the “liberal” group. The “liberal” subgroup consisted of patients who only met the criteria for PNS_1 only and not PNS_2 and/or PNS_H (i.e. all patients who met the criteria for PNS_2 or PNS_H automatically also met the criteria for PNS_1, but not all patients in the PNS_1 group met the criteria for PNS_2 or PNS_H). Of the 44 patients in the PNS_1 group, 23 patients met the “liberal” criteria. Therefore, 21 patients were left in the “stringent” group. Using the “liberal” group, repeated measures ANOVA were used to establish whether isolating this “liberal” group would impact the previous results with the entire PNS_1 group. Scores from the SOFAS scale were used as within subject variables (first assessment, month 12) and group (liberal, stringent) as the between subject variable. A main effect of time was observed (F _1,82_=48.089, p<0.001). However, although there was a significant [time x group] interaction using the entire PNS_1 cohort, isolation of the “liberal” group from this definition failed to reveal any significant [time x group] interactions (F_1,82_=0.879, p=0.351).

## Discussion

### Main findings

The main findings of this study suggest the prevalence of PNS in FEP varies depending on the definition being applied. More specifically, the prevalence of PNS was shown to be between 13 and 27%. Patients identified as having PNS (regardless of the definition) were consistently shown to have poorer functional outcome at month 12. However, all three PNS definitions demonstrated similar associations with functional outcome. Both PNS and non-PNS cohorts improved function over a 1-year period; however, the PNS group never met the criteria for “good functioning” according to mean SOFAS scores. Interestingly, when patients who met the criteria solely for our PNS_1 definition were extracted, this “liberal” definition did not show any significant associations with functional outcome at the one-year follow-up. The majority of patients met the PNS criteria due to clinically significant global scores on either the Avolition/Apathy or Anhedonia/Asociality domains of the SANS scale. Lastly, applying the proxy definition to identify FEP patients with DS resulted in a prevalence rate of 3%.

### Can the deficit syndrome criteria be applied in first episode psychosis?

The prevalence of primary enduring negative symptoms, or deficit syndrome in first episode patients has been estimated to be around 15% [46]. However, when applying the PDS to identify individuals with DS in a FEP cohort, the current findings suggested a prevalence of 3% when compared to 13-27% using the PNS criteria. Initially, the PDS was validated with a chronic schizophrenia outpatient cohort [9]. While DS only applies to schizophrenia spectrum disorder, the current study demonstrates that prominent and enduring negative symptoms or PNS impacting functional outcome include FEP patients with several primary diagnoses including schizoaffective, bipolar I with psychotic features and major depression with psychotic features. Accordingly, this may suggest that this subgroup of negative symptoms is relevant not only to patients with a diagnosis of schizophrenia. In fact, a diagnosis of schizoaffective disorder fell within the top three most common diagnoses for any of the three PNS definitions. It may be possible that when compared to DS, PNS is a more appropriate subgroup found in FEP patients with both affective and non-affective diagnoses.

It has been suggested that affective flattening and alogia (poverty of speech) are strongly associated and represent the “core negative symptoms” contributing to poor functional outcome [3,16,22]. In DS, affective flattening was previously shown to be significantly more severe in DS when compared to non-DS. The PDS case identification tool requires affective items to be subtracted from the blunted affect score of the PANSS or BPRS scales. In FEP patients, flat affect and alogia have not always been shown to be the most prominent negative symptoms [47]. Similarly, the results of the current study demonstrated low levels of the “diminished expression” subdomain of negative symptoms in the PNS cohort. It is plausible that these low levels of affective flattening in FEP greatly impact the prevalence of DS. More recently, the PDS formula was further altered to include both blunted affect and poverty of speech items [48]. Adding this second negative symptom would possibly further decrease the prevalence of DS in chronic schizophrenia and more so in a FEP population. Thus, in a group of FEP patients PNS, blunted affect and/or alogia do not seem to be the driving forces of these persistent symptoms and this may elucidate why the prevalence of DS was lower than previously documented [9].

### Influence of persisting negative symptoms on functional outcome

Similar to past findings in DS showing poorer functioning in DS when compared to non-DS [49], our results showed worse functional outcome at month 12 regardless of the PNS definition being applied. Several functional outcomes appear to be impacted by negative symptoms including psychosocial functioning, recreation, relationships, and occupational functioning [27,50,51]. An investigation by Milev et al. [27] assessed whether or not the severity of negative symptoms could predict functional outcome. Indeed, in comparison to positive and disorganized symptoms, negative symptom severity was shown to have the greatest predictability for poor psychosocial functioning. Concordantly, the current results demonstrated that applying the PNS_1 definition was sufficient to identify FEP patients with PNS at risk of poor functioning 12 months after entry into a FEP program.

Initial descriptions of schizophrenia included Kraepelin’s observation of avolition being prominent as a key symptom [52]. Accordingly, several studies have demonstrated more severe negative symptoms in the SANS subscales of anhedonia/asociality and avolition/apathy [16,25,53,54]. These SANS domains have also been associated with poor functional outcome, suggesting that the putative role of negative symptoms on functional outcome may be largely influenced by these domains. Similarly, our results showed greater mean scores on both these SANS domains as well as a greater number of FEP patients meeting the PNS criteria due to these SANS domains. Thus, the role of these two domains, which has been referred to as the “amotivation” subdomain of the SANS may play a pivotal role in PNS and its association with poor functional outcome [8].

Individually, the apathy domain of negative symptomatology has also been shown to contribute to poor functioning at year 1 [55,56]. However, given the content overlap observed when quantifying apathy and measuring functional outcome [57], this may have some influence on their relationship due to their tautology. This is a concern raised by previous authors [58] and may have been a limitation in the current study. Nonetheless, improving social and occupational functioning is a major objective in the treatment of FEP patients and a step towards recovery. Given the results of the current study, it may be more beneficial to identify individuals with PNS who are at a greater risk of functional decline by applying more stringent criteria. This will help build a stronger foundation for a more concise PNS definition.

### Secondary negative symptoms

Nineteen patients with PNS were removed from our analyses due to secondary negative symptoms. Secondary negative symptoms are thought to have a distinct etiology from that of primary negative symptoms [59]. Thus, to increase homogeneity of a cohort it is preferable to characterize both features of negative symptoms. However, investigations have not always made this distinction [60,61] - partly due to the difficulty in distinguishing primary from secondary negative symptoms [62-64]. It was suggested that secondary negative symptoms not responding to treatment should be included in the criteria for PNS [5]; albeit, the criteria proposed requires one to have minimal or no positive, depressive and extrapyramidal symptoms. It may be possible that patients with enduring secondary negative symptoms (due to positive symptoms not responding to treatment) may benefit specifically from interventions targeting PNS such as transcranial magnetic stimulation (TMS) [65-68]; however, this has not been empirically substantiated. Future studies should investigate the role these secondary negative symptoms have in PNS in order to provide a stronger rationale for including or excluding them from PNS.

### Characterization of the PNS cohort

Previous findings have documented longer DUP in patients with more prominent and enduring negative symptoms [3,24,63]. In a first episode cohort assessed during the first year of illness, DUP was also shown to predict PNS [3]. Furthermore, some studies have proposed that negative symptoms appear prior to the onset of positive symptoms, occurring in the prodromal period [69,70]. The current study did not replicate these findings; no significant group differences were found for DUP or length of the prodromal period. Regardless of the lower DUP and prodromal period, patients still met the criteria for PNS suggesting that there may be other factors contributing to PNS. Furthermore, at the first assessment, our PNS group had significantly worse negative symptoms when compared to the non-PNS group while positive symptoms were similar between groups. This may support the idea that more severe negative symptoms occurring earlier on have a significant contribution to residual negative symptoms. Hence, clinically significant negative symptoms at the onset of psychosis may be a strong indicator of PNS.

### Choosing a PNS definition

Choosing which PNS definition to employ may be dependent on the research question being asked. From an intervention perspective, the number of patients needs to be maximized to have a stronger conclusion determining the efficacy of a given intervention. Hence, applying our PNS_1 definition may be more appropriate. Interestingly, all patients who met the criteria for PNS_2 also met the criteria for either of the two domains of the hybrid definition (diminished expression or amotivation). Future research should focus on identifying the neurobiological and physiological determinants of PNS_1 and PNS_2 in order to determine whether they are distinct or share similarities. Furthermore, as suggested by Buchanan [5], it may be beneficial to include patients with persistent secondary negative symptoms that have not responded to treatment when employing this particular research question.

#### Limitations

Some studies have suggested that a follow-up of two or more years may be more appropriate when exploring symptoms in FEP [27,71]. Our study had a one-year follow up and this may have been a limitation in terms of understanding the trajectory of PNS. A 5-year follow up may have helped us better delineate the course of PNS in our FEP cohort.

## Conclusions

Persistent negative symptoms are present in about 27% of FEP patients. Applying either of our PNS definitions for identifying PNS is a feasible method for identifying patients with PNS at risk of poor functioning. However, the definition being employed should depend on the research objectives. Given the association between PNS and poor functional outcome 12 months after entry into our treatment program, it is highly recommended to identify PNS within the first year of illness. When compared to the PDS, using a PNS criteria may be more applicable to a FEP cohort to identify enduring negative symptoms. As it has been stated ad nauseam, we need to standardize how to define PNS in order to obtain a better understanding of these symptoms. Further longitudinal, rather than cross-sectional studies on the development and treatment of PNS in a FEP population are warranted.

## Abbreviations

BPRS: Brief Psychiatric Rating Scale; CDSS: Calgary Depression Scale for Schizophrenia; CORS: Circumstances of Onset and Relapse Schedule; DS: Deficit schizophrenia; DUI: Duration if Untreated Illness; DUP: Duration of Untreated Psychosis; FEP: First Episode Psychosis; IRAOS: Interview for the Retrospective Assessment of Schizophrenia; NIMH: National Institute of Mental Health; PNS: Persistent Negative Symptoms; PAS: Premorbid Adjustment Scale; PANSS: Positive and Negative Syndrome Scale; PDS: Proxy for the Deficit Syndrome; SCID-IV: Structured Clinical Interview for DSM-IV; SANS: Scale for the Assessment of Negative Symptoms; SAPS: Scale for the Assessment of Positive Symptoms; SDS: Schedule for Deficit Syndrome; WAIS: Wechsler Adult Intelligence Scale.

## Competing interests

The authors declare that they have no competing interests.

## Authors’ contributions

CLH drafted the first manuscript. RJ, AKM and ML contributed to the design of the study. AKM and JM managed all patient recruitment and clinical assessments. CLH and MB carried out the data analysis. All authors contributed significantly to the interpretation of the data as well as having read and approved the final manuscript.

## Pre-publication history

The pre-publication history for this paper can be accessed here:

http://www.biomedcentral.com/1471-244X/12/224/prepub
